# Non-surgical Retrieval of Dislodged or Misplaced Intravascular/Endoluminal Foreign Objects

**DOI:** 10.7759/cureus.3931

**Published:** 2019-01-21

**Authors:** Muhammad Danish Barakzai, Tanveer U Haq, Kumail Khandwala

**Affiliations:** 1 Radiology, Aga Khan University Hospital, Karachi, PAK

**Keywords:** retrieval, vascular, malposition, foreign body, interventional radiology, misplaced

## Abstract

Interventional procedures involving the use of intravascular or endoluminal objects have rapidly increased over the years with advancements in minimally invasive techniques. These foreign objects such as endovascular coils, guidewires, and endoluminal catheters, if lost or malpositioned, are a potential threat, which can result in complications such as embolization, perforation, infections, and arrhythmias. Therefore, timely removal of these foreign bodies is essential. In this technical report, we have described our experience with different scenarios in which percutaneous interventional techniques for retrieval of such foreign bodies were performed at our institute.

## Introduction

Minimally invasive and interventional techniques involving the implantation and use of intravascular objects have rapidly increased over the years [1], and simultaneously, there is an increase in the number of such objects that become malpositioned or misplaced. These foreign objects are a potential threat that can result in embolization, perforation, infections, and arrhythmias [2]. Therefore, timely and effective removal of these foreign bodies is essential. The spectrum of iatrogenic endoluminal foreign objects has broadened to include items such as embolization coils and endoluminal catheters, and most of the studies and case reports focus on intravascular wire retrieval [3]. Appropriate tools, expertise, and prompt actions are required while retrieving a lost object using minimally invasive techniques.

## Technical report

We report four different scenarios wherein percutaneous interventional techniques for retrieval of foreign bodies were performed at our institute. Formal ethical review was taken from the Ethics Review Committee at the Aga Khan University Hospital, Karachi prior to conducting the study. Informed consent was taken from each patient before performing the procedures.

Patient 1

A 35-year-old male patient presented to the emergency room (ER) with multiple episodes of hematemesis and melena. Upper gastrointestinal (GI) endoscopy showed a large duodenal ulcer with a clear base and a visible oozing vessel. Argon plasma coagulation (APC) was performed; however, bleeding persisted. Injection of adrenalin in aliquots at the bleeding site was given; however, bleeding could not be controlled. Multiple small healed ulcers were also seen at the antrum. The patient was subsequently intubated and managed along the lines of hemorrhagic shock and disseminated intravascular coagulation (DIC). Resuscitation was done along with massive transfusion protocol. Omeprazole infusion was continued and norepinephrine started. 

The patient was shifted to the interventional radiology (IR) suite immediately. A 4 French (Fr) vascular access femoral sheath was placed followed by the insertion of a 4 Fr C1 catheter. Angiogram of the celiac and superior mesenteric artery showed significant spasm. Active extravasation of contrast was noted from the gastroduodenal and inferior pancreaticoduodenal arteries.

**Figure 1:**
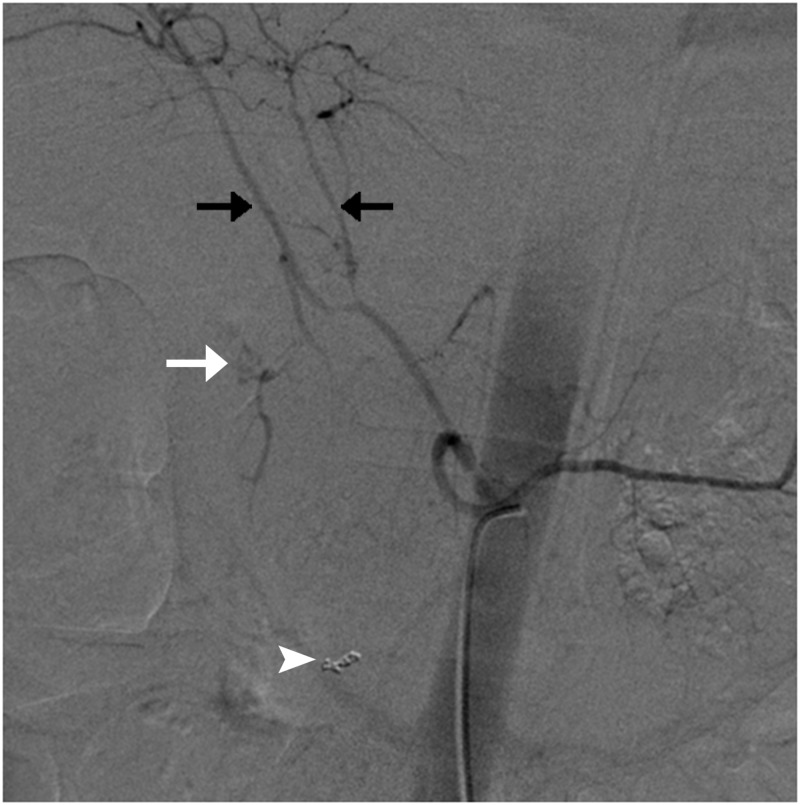
Angiogram of celiac artery showing active extravasation of contrast from gastroduodenal artery (white arrow); coils in the inferior pancreaticoduodenal artery (white arrowhead); the right and left hepatic arteries are also seen (black arrows).

A Progreat microcatheter (2.7 Fr, catheter 130-cm long, with a 0.021-inch wire) was used to selectively cannulate these arteries. The inferior pancreaticoduodenal artery was successfully embolized first with a coil (Figure 1).

**Figure 2:**
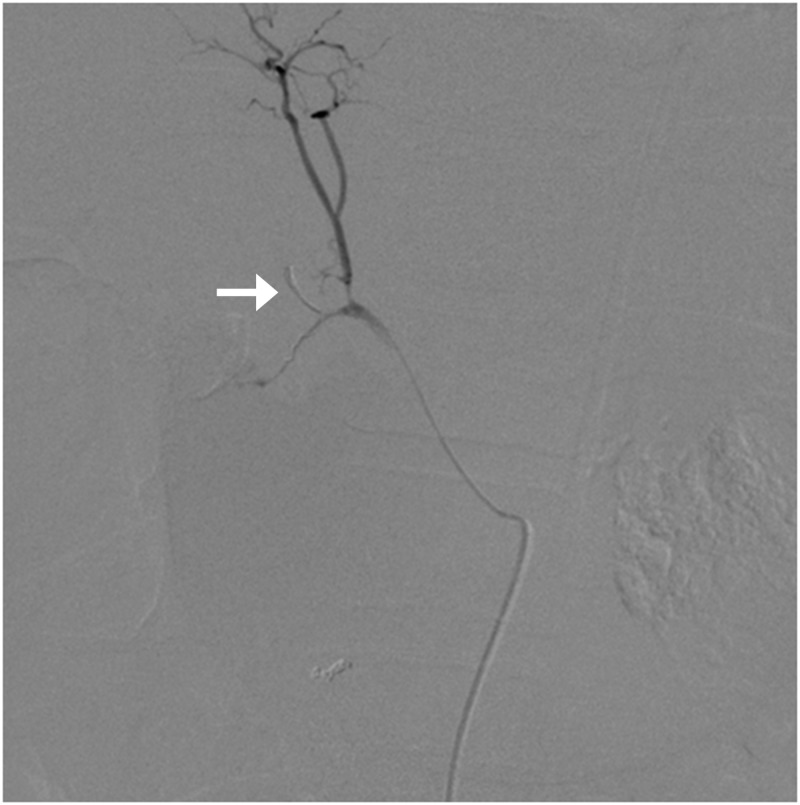
While embolizing the gastroduodenal artery, a part of the coil got dislodged into the right hepatic artery (arrow) resulting in its occlusion.

While placing the FIB2x25 pushable coil to embolize the gastroduodenal artery, the coil got dislodged into the right hepatic artery (Figure 2).

**Figure 3:**
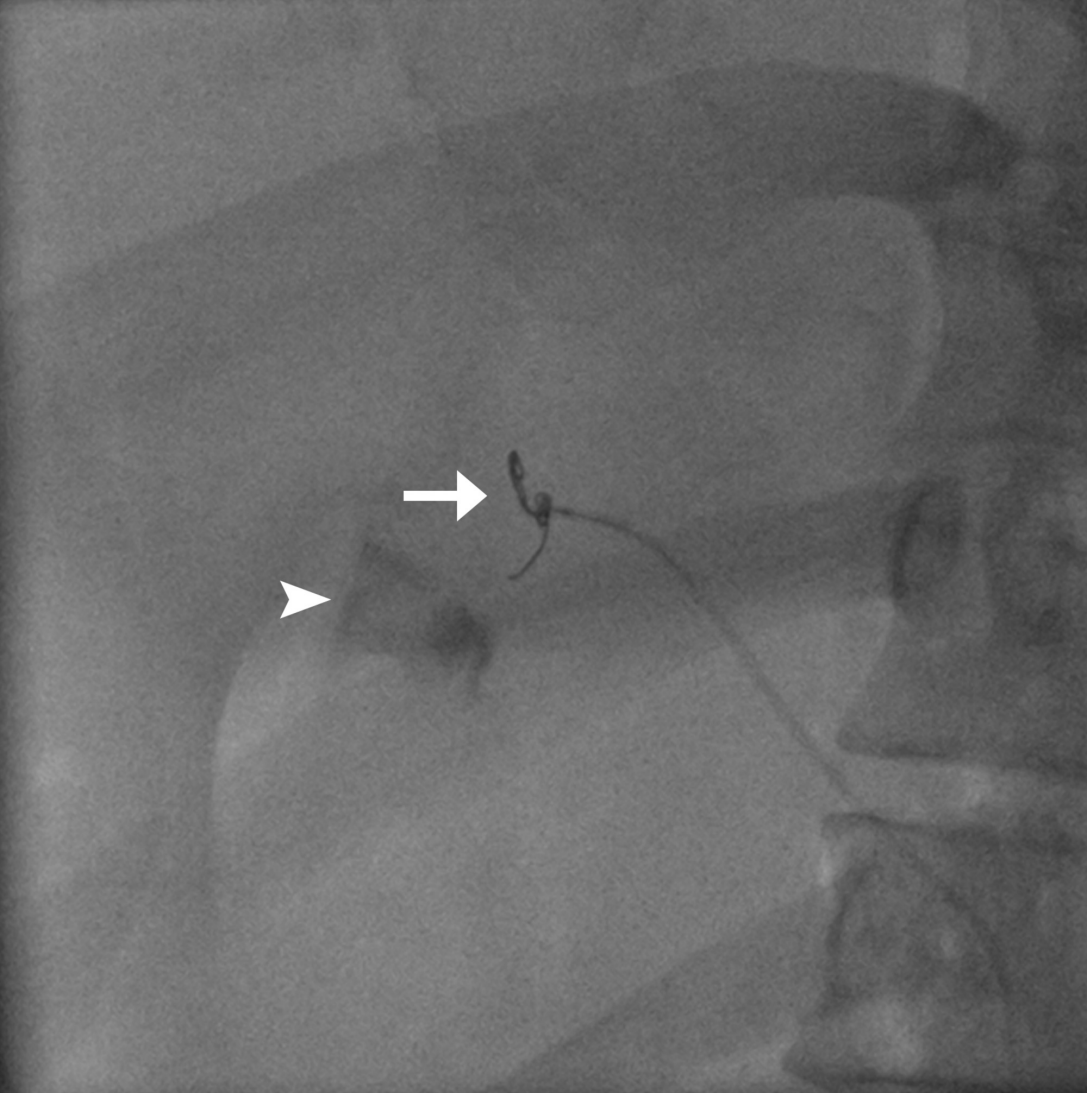
Dislodged coil ensnared with gooseneck microsnare (arrow); active extravasation from the gastroduodenal artery (arrowhead).

**Figure 4:**
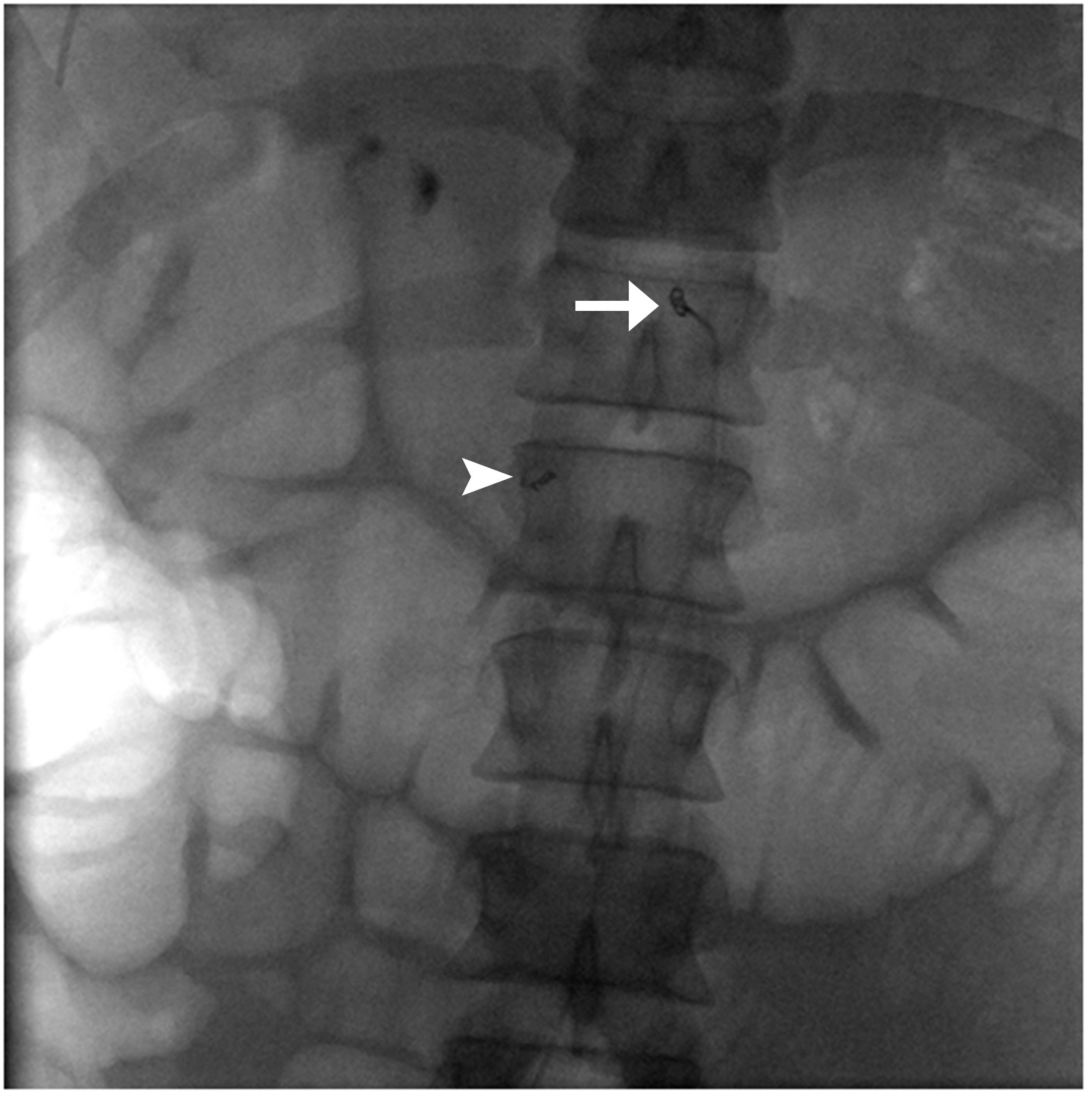
Trapped coil in the aorta (arrow); coil in the inferior pancreaticoduodenal artery (arrowhead).

**Figure 5:**
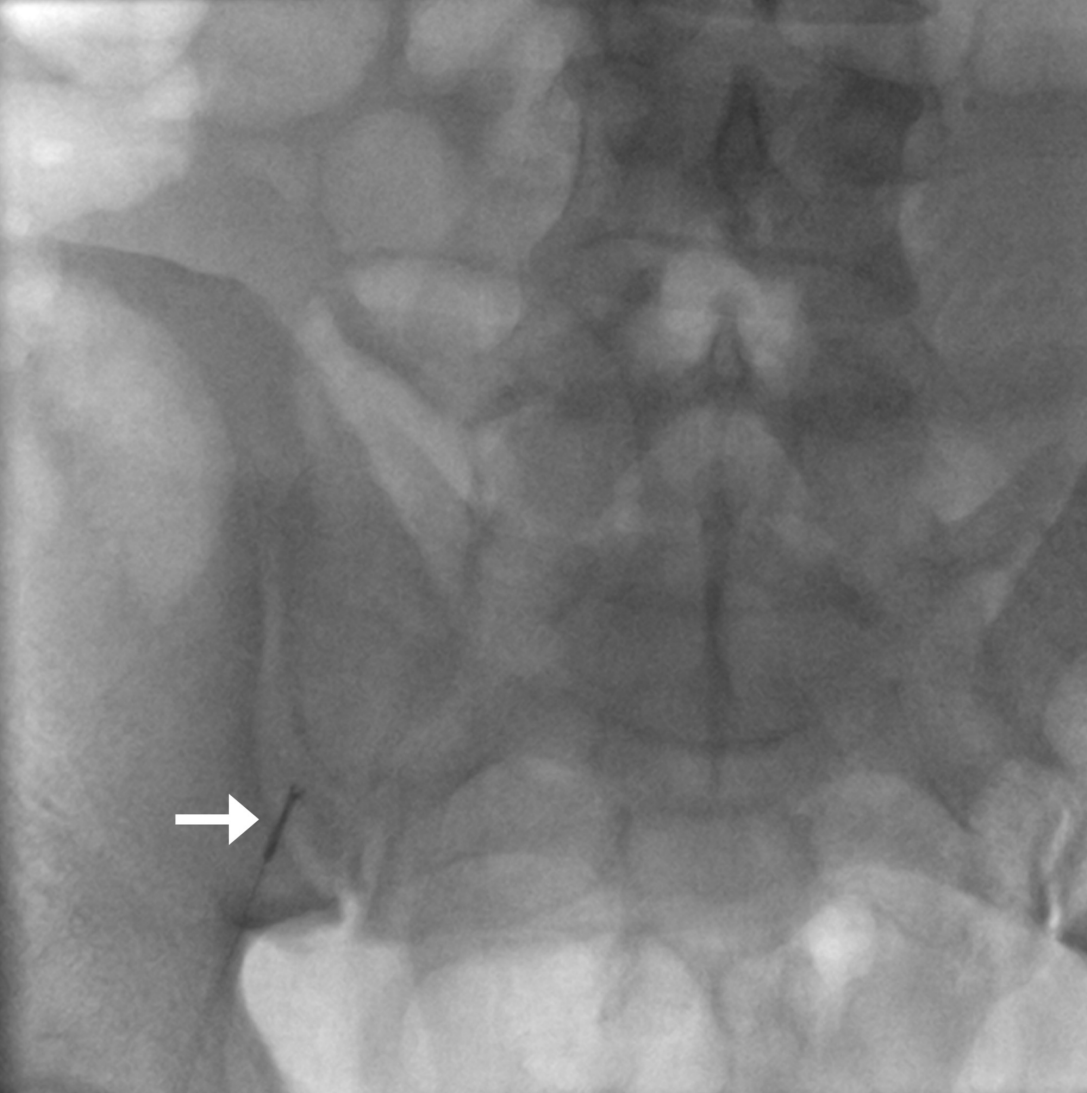
Fluoroscopic spot image showing coil being withdrawn from the right femoral sheath approach.

A microsnare (175-cm long, 4-mm loop, 0.18 inch) was used to catch the lost coil and it was retrieved percutaneously through the right femoral sheath (Figures 3-5). Subsequently, the gastroduodenal artery was embolized using multiple coils and histoacryl glue.

Patient 2

**Figure 6:**
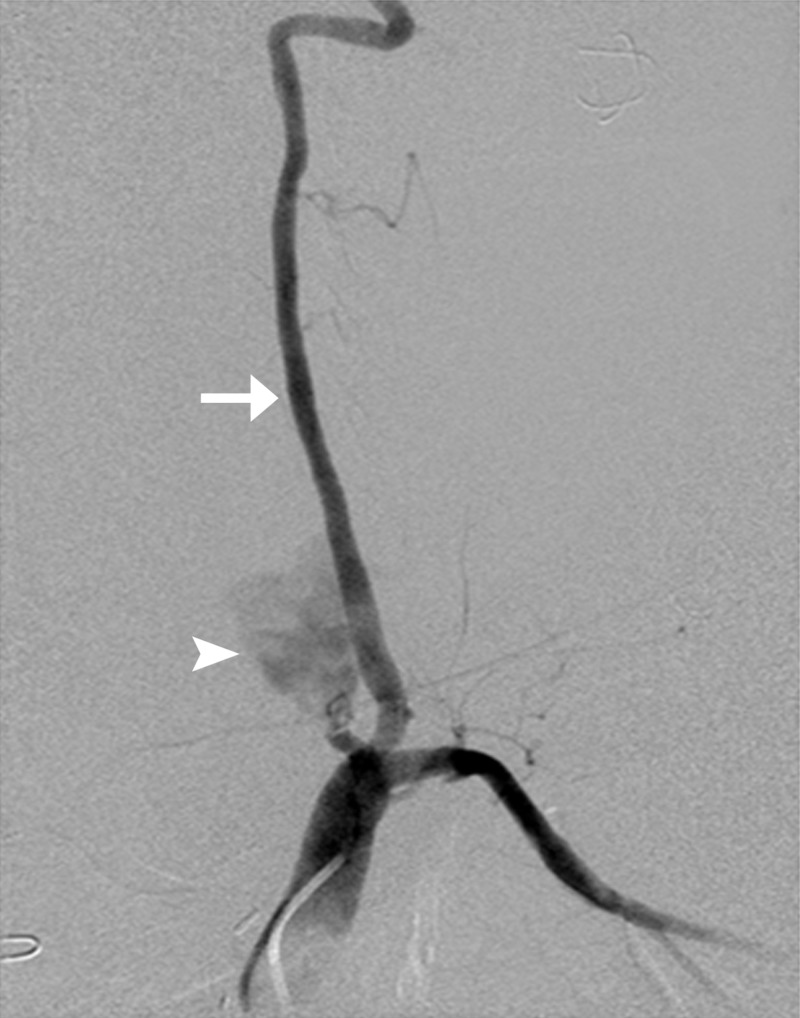
Angiogram showing active extravasation of contrast from left thyrocervical trunk (arrowhead). The left vertebral artery (arrow) appears normal.

A 40-year-old male presented in the ER with a history of multiple gunshots to the head, neck, thorax, abdomen, and the right lower limb. Computed tomography (CT) scan was done which showed active extravasation from the left thyrocervical trunk, inferior thyroid artery, and the left facial artery which was confirmed on angiography (Figure 6).

**Figure 7:**
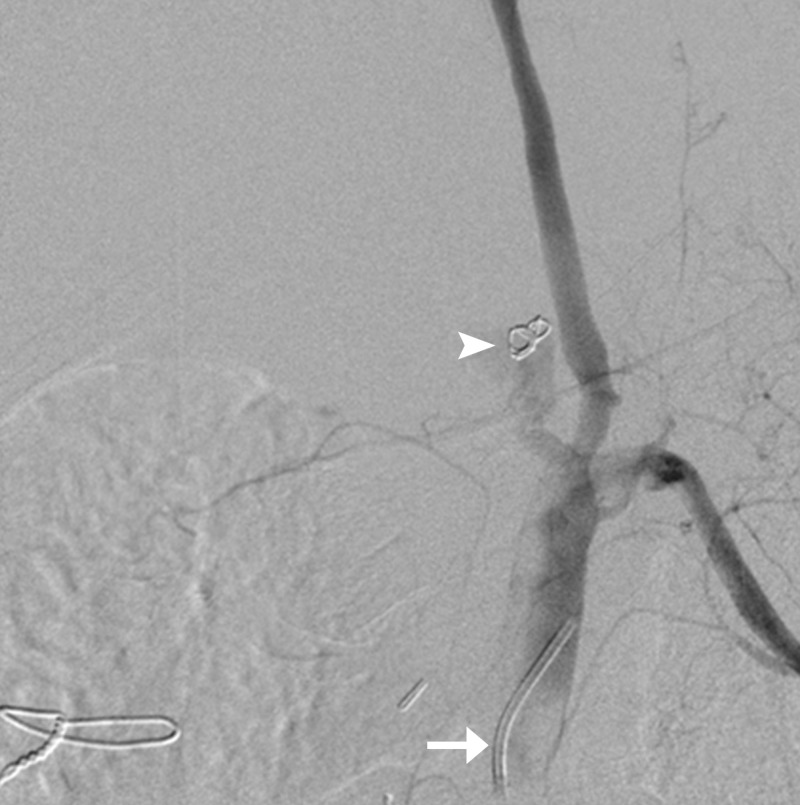
A 4 French H1 catheter in the left subclavian artery (arrow); coil within the extravasating thyrocervical trunk (arrowhead).

**Figure 8:**
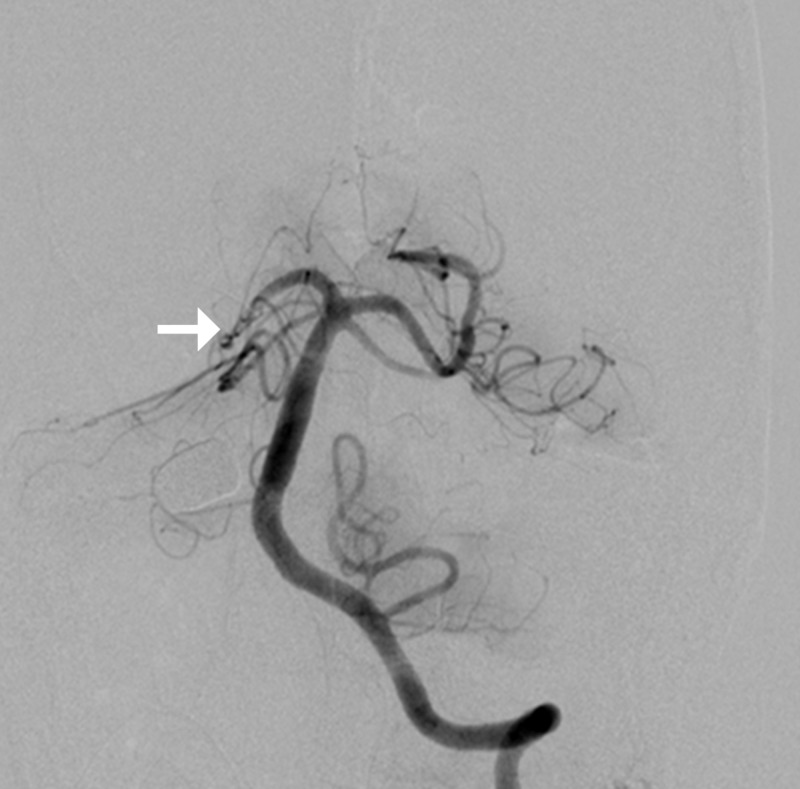
Left vertebral angiogram showing coil dislodged into the right posterior cerebral artery (arrow). The coil is occluding the distal flow in this vessel.

The patient was planned for angioembolization. During the procedure, while embolization of left thyrocervical trunk, the coil got dislodged into the right posterior cerebral artery and occluded its distal flow (Figures 7-8).

**Figure 9:**
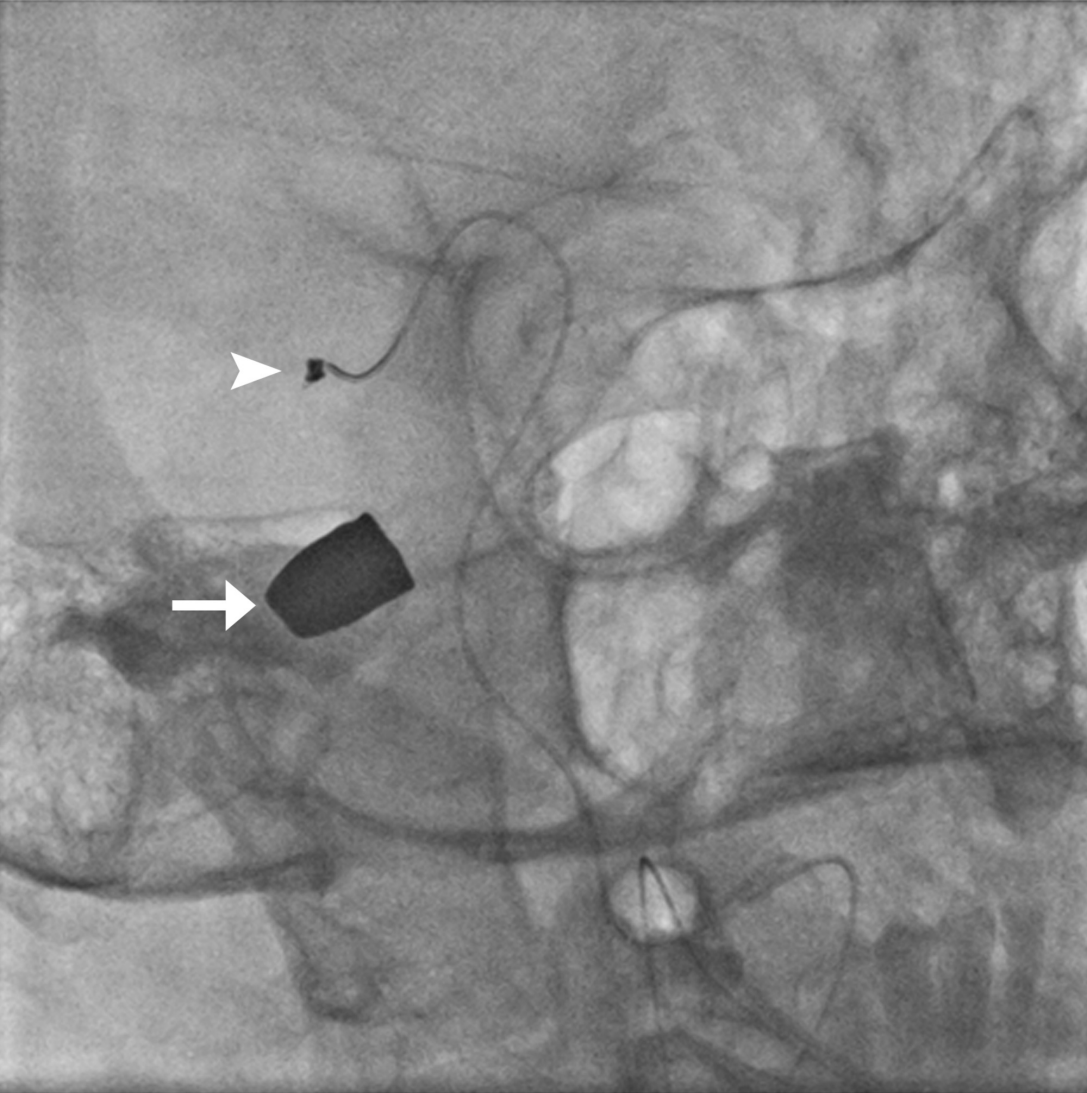
Gooseneck microsnare holding the coil (arrowhead). A bullet (arrow) is visualized above the clivus on the right side.

**Figure 10:**
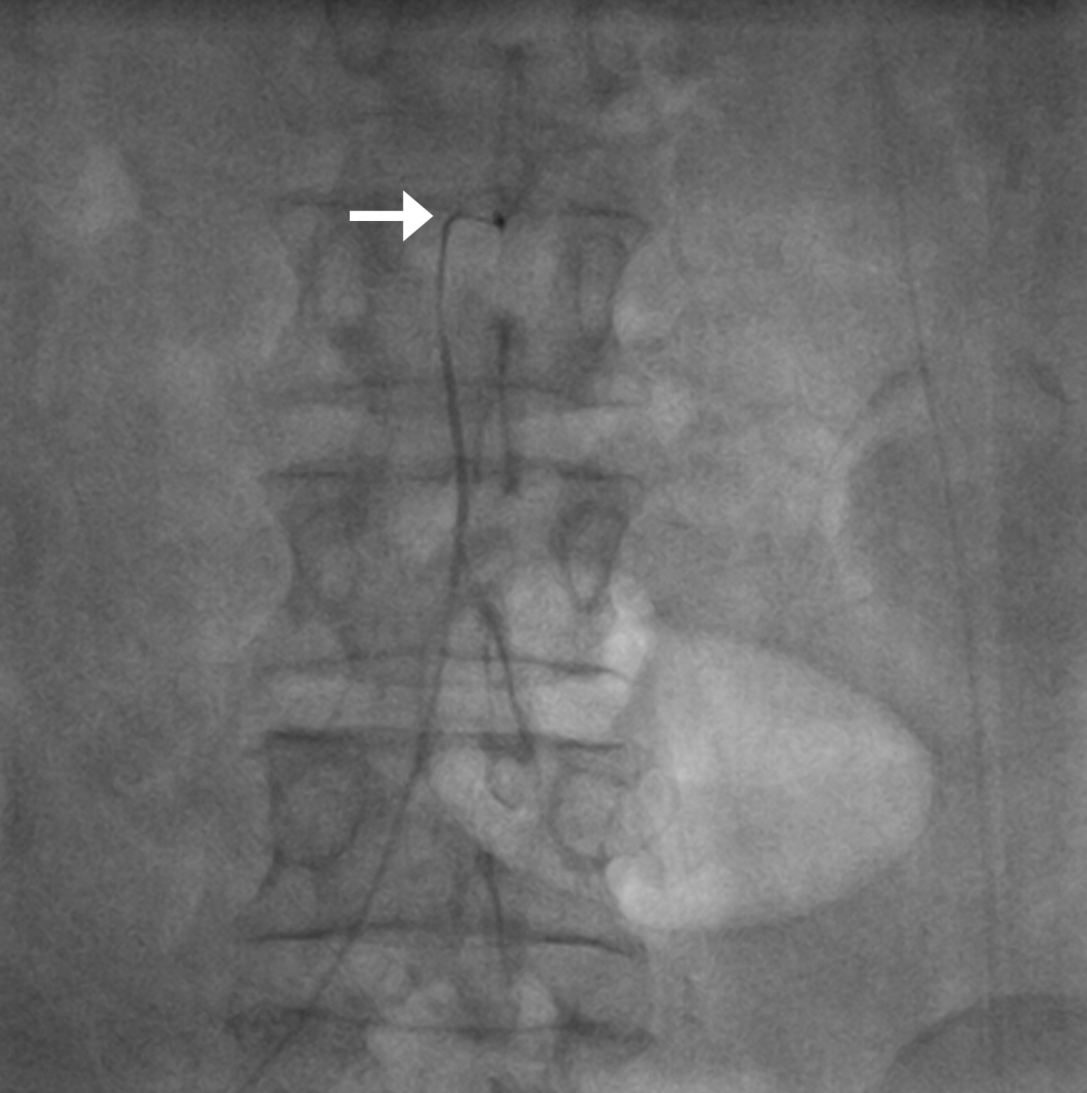
Trapped coil and microsnare in the abdominal aorta

A microsnare (175 cm length, 4-mm loop, 0.18 inch ) was used to catch the lost coil and it was successfully retrieved off the right femoral sheath (Figures 9-10).

**Figure 11:**
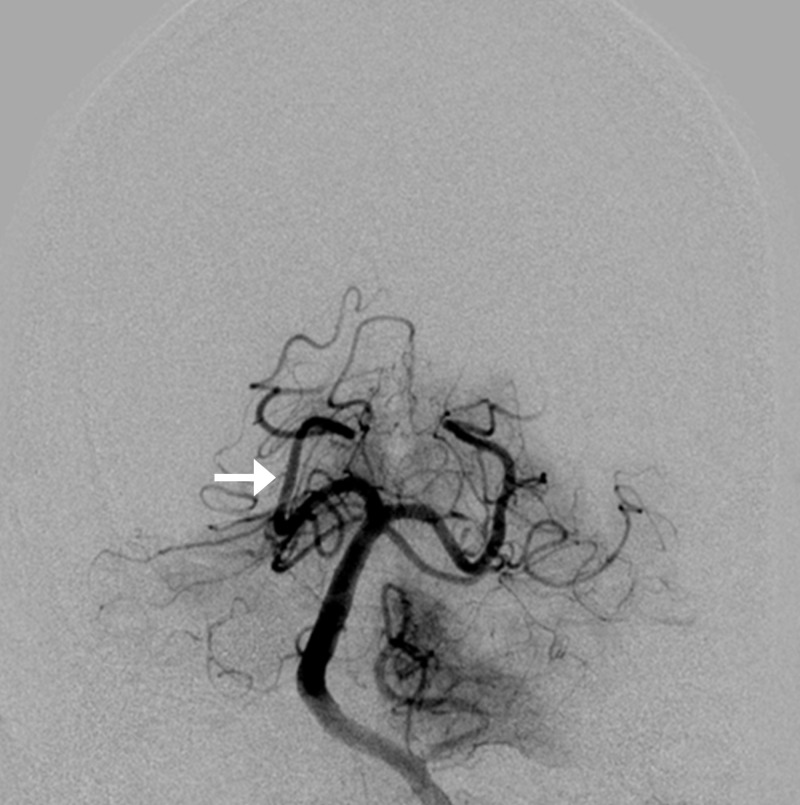
Left vertebral angiogram post-coil removal showing normal flow in the right posterior cerebral artery (arrow)

Post-retrieval angiogram of the vertebral artery showed normal flow in the right posterior cerebral artery (Figure 11).

Patient 3

**Figure 12:**
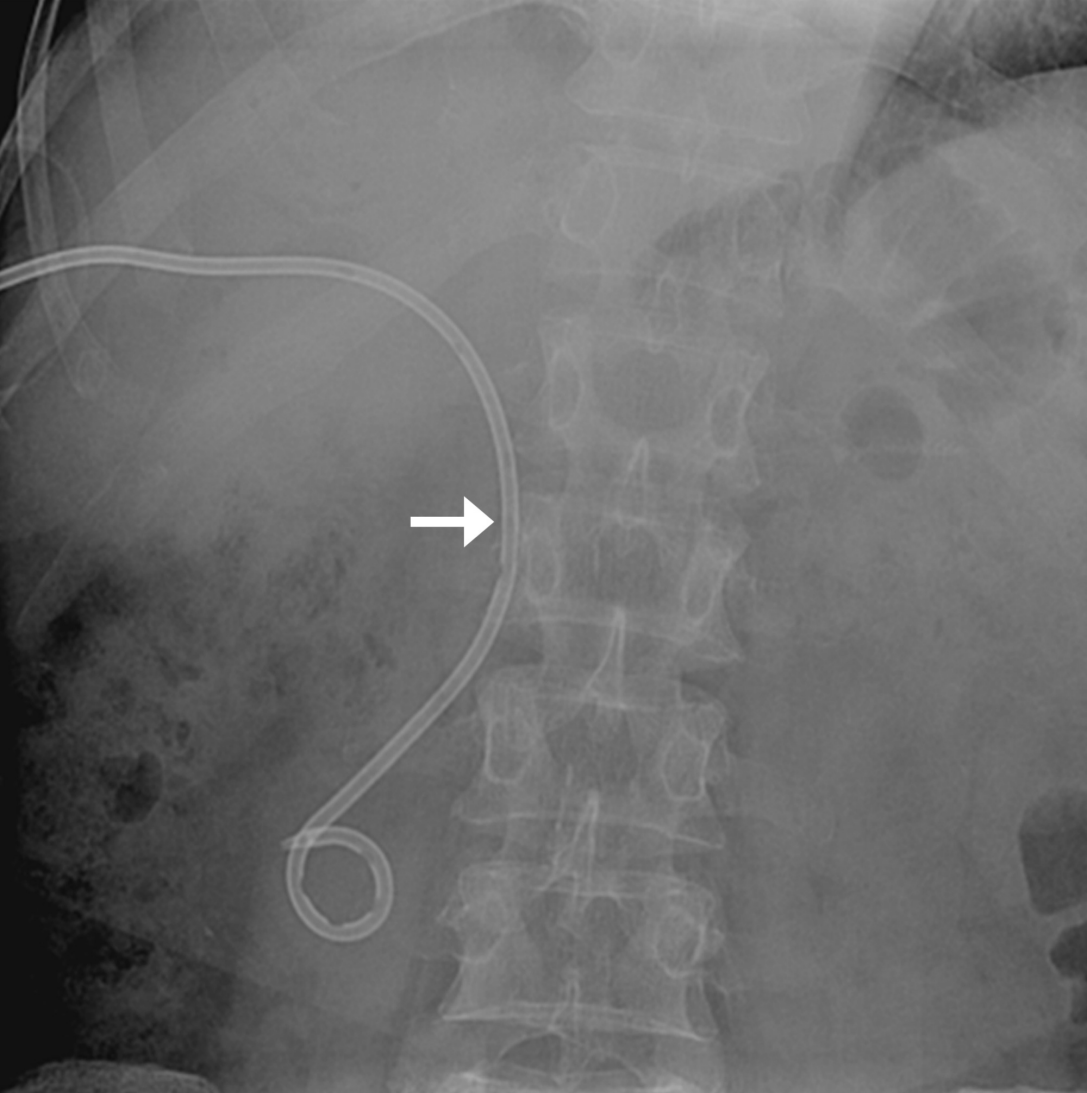
Fluoroscopic spot image showing internal/ external biliary drain in place (arrow).

A 65-year-old male, whose status was post-Whipple procedure, developed jaundice after five months of the surgery. Liver function tests (LFTs) showed total bilirubin level of 5.3 mg/dL, direct bilirubin level of 4.1 mg/dL, and an indirect bilirubin level of 1.2 mg/dL, gamma-glutamyl transferase (GGT) level of 1012 U/L, and alkaline phosphatase (AP) level of 460 U/L. Cholangiogram was performed which showed mild dilatation of intrahepatic bile ducts and a stricture at the hepaticojejunostomy anastomotic site. Multiple filling defects were seen within the intrahepatic ducts especially in the left main hepatic duct representing stones. Following balloon cholangioplasty, an 8 Fr locking pigtail catheter was placed across the stricture into the bowel as an internal-external biliary drain (Figure 12).

**Figure 13:**
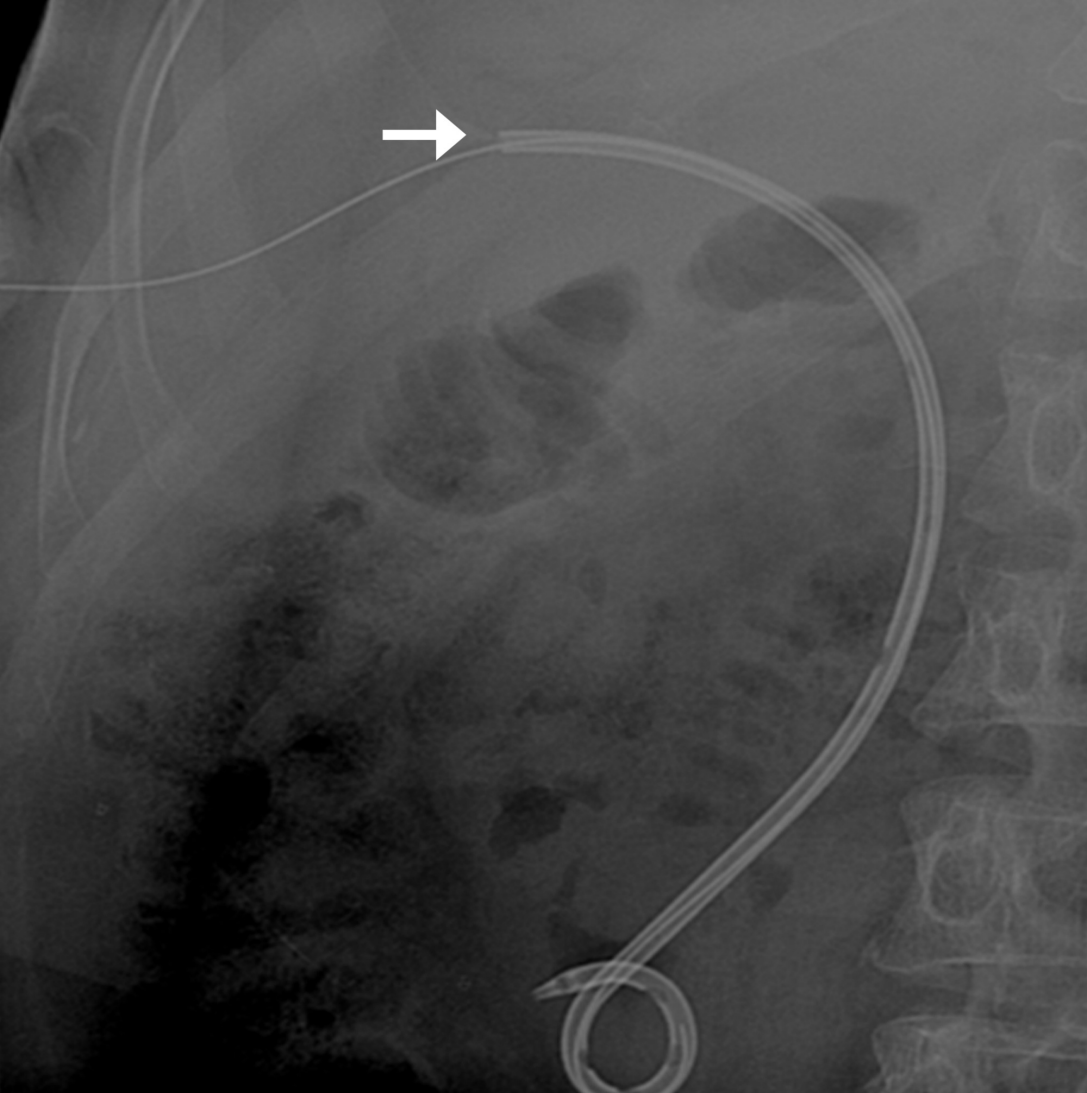
Fluoroscopic spot image showing glidewire cannulating the dislodged catheter (arrow)

**Figure 14:**
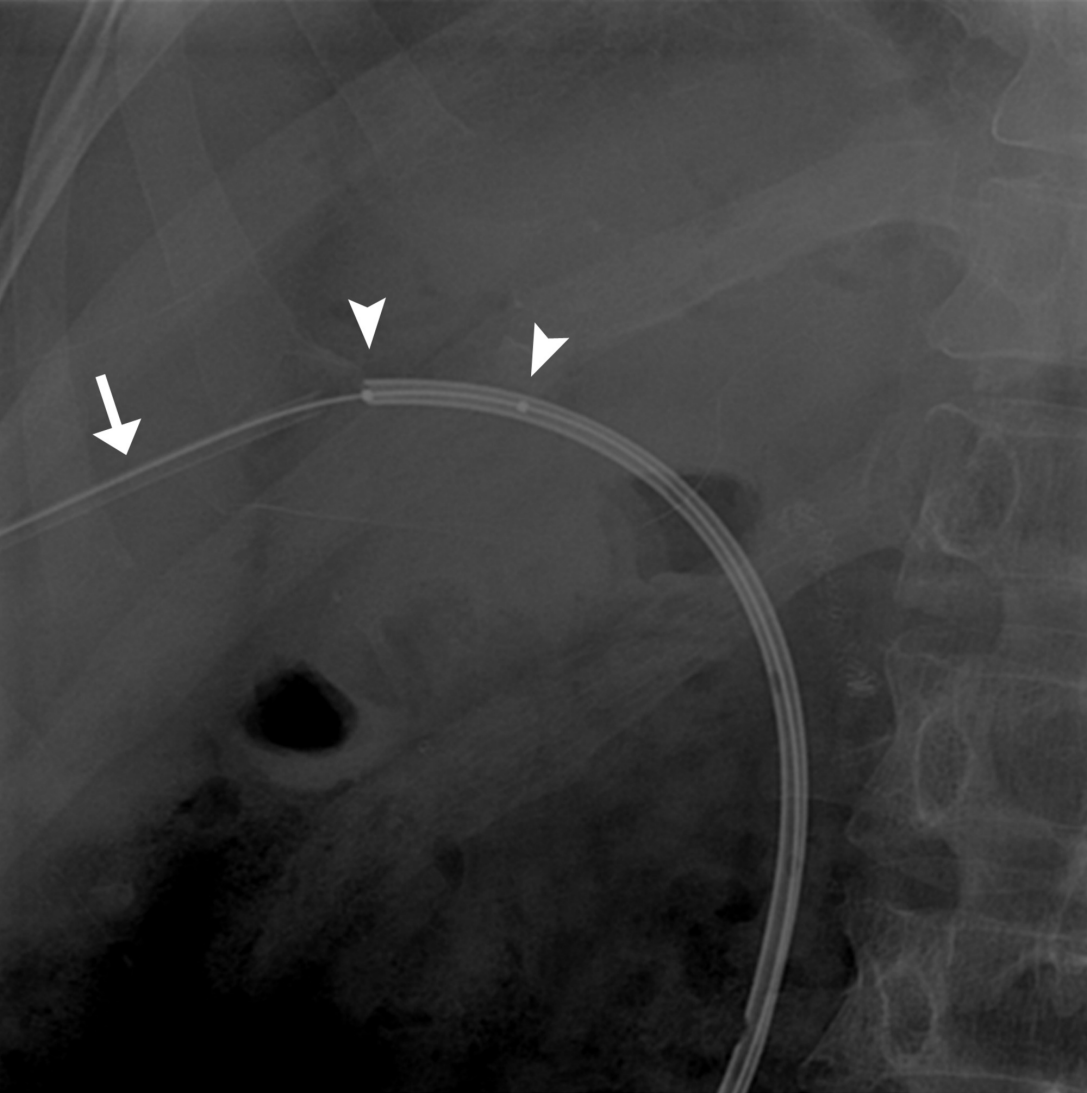
Fluoroscopic spot image showing 7 French sheath (arrow) and balloon deployed in the catheter (arrowheads).

**Figure 15:**
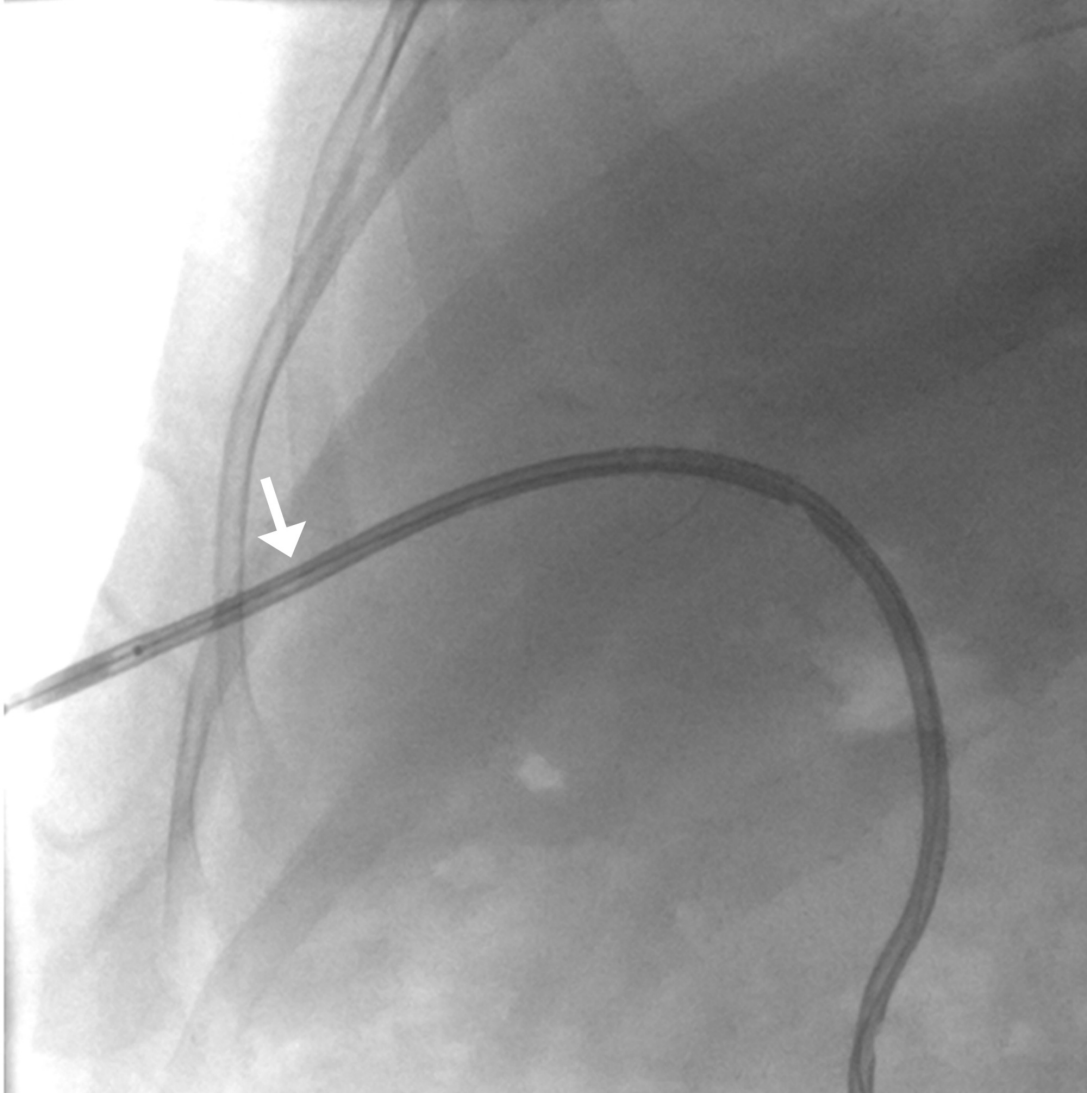
Fluoroscopic spot image showing retrieval of pigtail catheter

Initially, the patient had improvement in liver function but again came with deranged LFTs in increasing trend. He was planned for re-cholangioplasty and placement of 12 Fr pigtail catheter. During removal of the previously placed pigtail catheter, it slipped into the intrahepatic ducts. A glidewire was placed to cannulate the misplaced pigtail and over the wire, a 7 Fr sheath was placed into the tract. A 3 mm x 2 cm over the wire balloon was used to extract the catheter (Figures 13-15).

Subsequently, a new 12 Fr pigtail catheter was successfully deployed after cholangiopasty.

Patient 4

A 45-year-male came to the ER with fever for six days, vomiting for two days, and three episodes of per oral bleeding. The patient had a history of exposure to cattle and ticks. Laboratory workup showed a positive polymerase chain reaction (PCR) for Congo virus and deranged LFTs. Shortly after, the patient developed respiratory distress because of which he was intubated and shifted to the intensive care unit (ICU) for observation. Meanwhile, a multi-lumen central line (arrow, 7 Fr, 16 cm length with 0.32-inch guidewire) was placed in the ICU via the right femoral approach.

**Figure 16:**
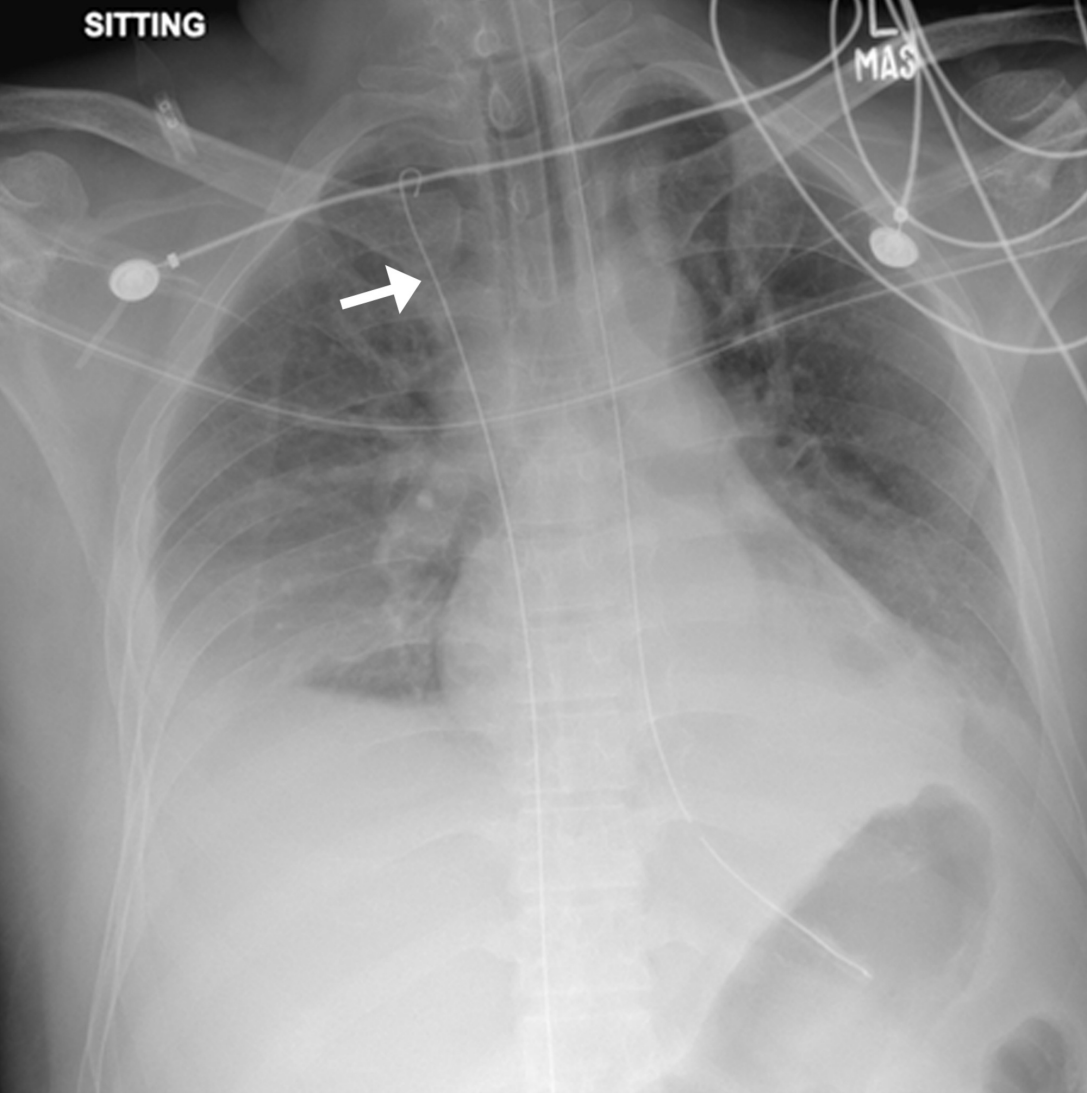
Post-procedural portable chest radiograph showing trapped guidewire (arrow); nasogastric tube and endotracheal tube also seen in place

After the procedure, while collecting instruments, the guidewire was missing. An X-ray confirmed that the guidewire was lodged within the IVC, crossing the heart with its tip reaching the right internal jugular vein (Figure 16).

The IR team was taken on board to remove the wire. The patient was shifted to the IR suite with all the precautions. Initial fluoroscopic spot confirmed the exact position of the wire. 

**Figure 17:**
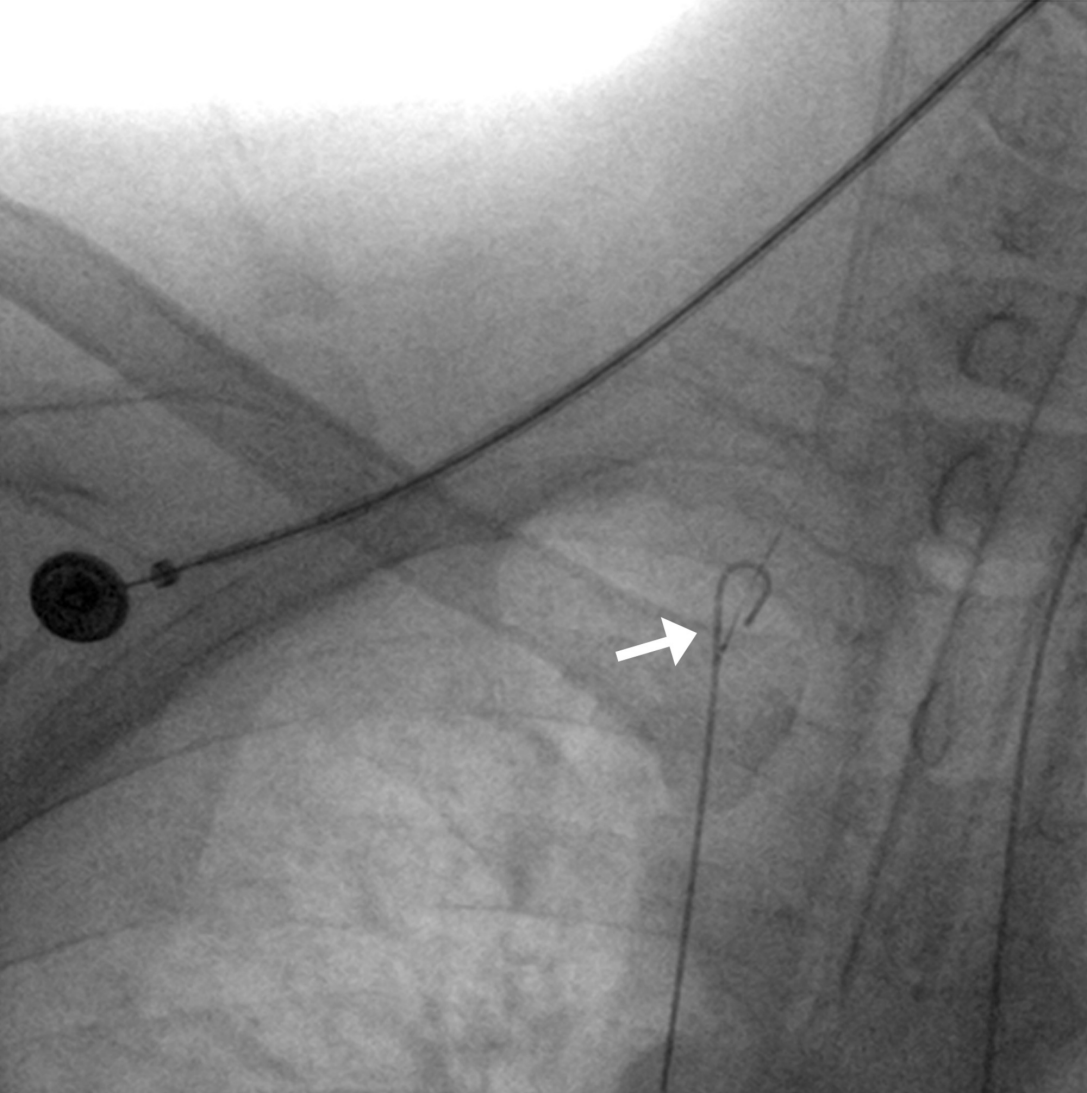
Fluoroscopic spot image shows snared J-tip of guidewire (arrow) at the level of the right internal jugular vein.

**Figure 18:**
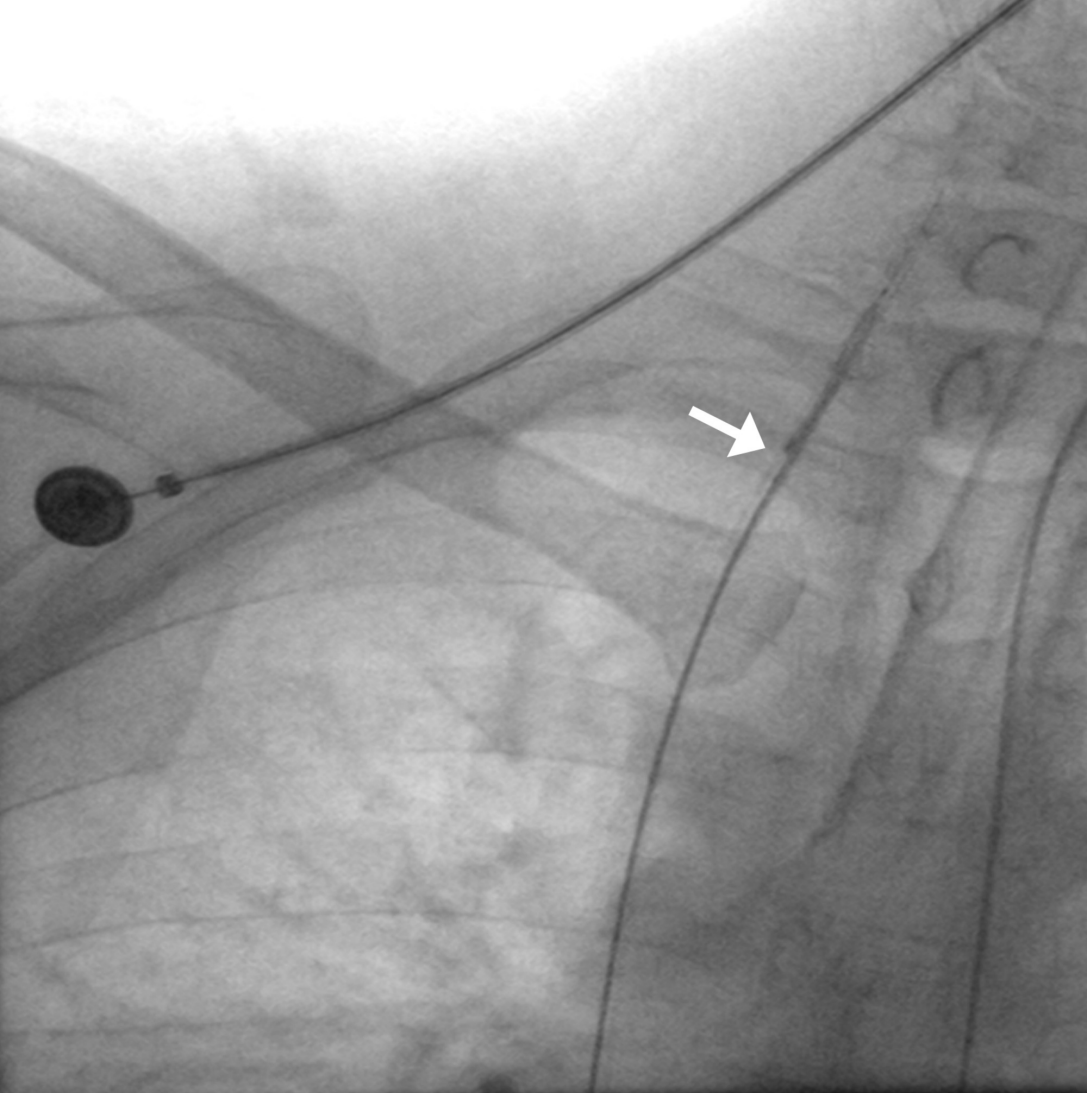
The tip of the trapped 0.18-inch wire is within the sheath (arrow).

Subsequently, a 7 Fr sheath was placed in the right jugular vein, and a microsnare (175-cm long, 4-mm loop, 0.18 inch) was used. Multiple attempts were made to get hold of the J-tip but were unsuccessful. The distal straight end of the wire was targeted to snare. Once in position, gradually the snare was retracted under fluoroscopy to reach the proximal J-tip. The J-tip was snared and the wire was removed from the sheath (Figures 17-18).

## Discussion

New endovascular materials and devices require constant education and updated expertise for the intervention. Similarly the technical proficiency required to deal with dislodged or misplaced endoluminal material also needs enhancement [3]. Foreign bodies should be removed as soon as possible because of above mentioned complications. Major complications after embolization of these particles are reported as high as 71% [4].

Dotter et al. in the first review of the percutaneous retrieval of intravascular foreign bodies refer exclusively to catheters and wire fragments, but now the spectrum of intravascular devices and objects has broadened significantly to include items such as vena cava filters, embolization coils, and endovascular stents [5]. For most of the cases, percutaneous treatment of intravascular foreign bodies continues to be safely and effectively applied in numerous patients [3]. 

Retrieval tools have transformed and evolved in recent decades. Dormia basket was used in the early 1980s, while gooseneck snare or grasping forceps is mostly used nowadays [3]. The Amplatz gooseneck snare is, by far, the most often used device for the retrieval of intravascular foreign objects. The loops and shaft feature the shape memory and superelastic properties of nitinol, and so they retain their shape and are extremely kink-resistant [1,6]. It is also the most effective method with high success and fewer complication rates [1]. Additionally, the snare is capable of developing a variable amount of force, and foreign bodies can be easily compressed.

In a study by Gabelmann et al, the most frequently used device was a combination of an angled snare and a multipurpose catheter with a hook configuration. This made it possible to retrieve foreign objects even in difficult locations because of the two-fold angles of the gooseneck and a multipurpose catheter. They reported a success rate of 91.1% [7]. Another study shows a technical success rate in 19 out of 24 cases (79.2%) [8]. Therefore, the goose snare is a highly efficient atraumatic instrument. The relevance of these methods cannot be underestimated. Numerous available rescue devices to retrieve lost objects and the creative usage of wires and catheters in different situations make difficult cases safer and easier to perform. 

In our experience, percutaneous treatment of intravascular foreign bodies was successful in all four patients. The microsnare was also the most valuable tool for the retrieval of endovascular and endoluminal foreign body devices. Because of its inherent flexibility and preformed intravascular configuration, it has been possible to snare coils within small-sized vessels. No post-retrieval complications, such as damage to the vessel wall, were noted in our patients. However, previous studies have shown minor complications such as groin hematoma cardiac arrhythmias, ventricular or vessel perforation, artery spasm, thrombosis, and injury to the vessel at the puncture site [7,9-10]. The most important limitation was the need of fluoroscopy, which has an inherent risk of radiation exposure to the patient. The biggest drawback we felt is the cost of the snare, for which it has to be re-sterilized and autoclaved, and so that it could be reused in our setting.

## Conclusions

In conclusion, endovascular retrieval of lost or misplaced foreign devices, particularly with the Amplatz goose neck snare, is highly effective. On the basis of this single-center technical report of four cases, this method has proven to be safe and should be considered the tool of choice for the removal of intravascular/endoluminal foreign objects.
